# Assessing the performance of TRX and DUF148 antigens for detection of prepatent Guinea worm (*Dracunculus medinensis*) infection in dogs

**DOI:** 10.3389/fpara.2025.1699367

**Published:** 2025-11-21

**Authors:** Hassan Hakimi, Pabasara Weerarathne, Meriam N. Saleh, Raquel R. Rech, Richard Ngandolo Bongo Nare, Philip Ouakou Tchindebet, Sidouin K. Metinou, Jessica M. van Loben Sels, Lucienne Tritten, Guilherme G. Verocai

**Affiliations:** 1 Department of Veterinary Pathobiology, College of Veterinary Medicine and Biomedical Sciences, Texas A&M University, College Station, TX, United States; 2 Department of Diagnostic Medicine and Pathobiology, College of Veterinary Medicine, Kansas State University, Manhattan, KS, United States; 3 Institut de Recherche en Élevage pour le Développement, Afrique One Aspire, N’Djamena, Chad; 4 Programme National d’Éradication du Ver de Guinée – Tchad, Ministère de la Santé, N’Djamena, Chad; 5 The Carter Center, National Guinea Worm Eradication Program – Chad, N’Djamena, Chad; 6 Guinea Worm Eradication Program, The Carter Center, Atlanta, GA, United States; 7 Institute of Parasitology, Faculty of Agricultural and Environmental Sciences, McGill University, Sainte-Anne-de-Bellevue, QC, Canada

**Keywords:** Guinea worm, eradication, dog infections, DUF148, serology

## Abstract

**Introduction:**

Guinea worm (GW) is a nematode that causes a neglected tropical disease that is targeted for eradication. GW emergence in animals, particularly dogs, has hampered eradication efforts. Currently, there is no method for diagnosing GW infection in animals during the prepatent period. Previous work has identified two immunoreactive antigens, TRXL-1 (TRX) and DUF148.

**Methods:**

This study developed and assessed the performance of an indirect ELISA using these antigens.

**Results:**

Using serum samples from experimentally exposed dogs, TRX and DUF148 showed reactivity at 9- and 11-weeks post-exposure, respectively. These antigens were further assessed using sera of dogs from GW-endemic villages in Chad (n=47) and shelter dogs from the non-endemic United States (n=492). DUF148 showed better reactivity and sensitivity of 76.6.% in detecting GW infection in prepatent sera compared to TRX. However, DUF148 cross-reacted with a *Brugia pahangi* experimental infection serum sample and several shelter dog sera. To mitigate this cross-reaction, we produced 3 peptides that spanned different regions of DUF148. Peptide 3 from the C-terminal was more reactive with prepatent sera and had a sensitivity of 83%; however, the specificity was not superior to whole antigen.

**Discussion:**

Our findings could facilitate the development of diagnostic methods for early detection of GW infection in dogs in endemic countries.

## 1 Introduction

Guinea worm (GW; *Dracunculus medinensis*) is a parasitic nematode that causes dracunculiasis, or GW disease, a debilitating neglected tropical disease. Humans acquire the parasite by ingesting infected cyclopoid copepods in water. The copepod serves as the intermediate host and contains the infective third-stage GW larvae (L3) (Cairncross et al., 2002; Ruiz-Tiben and Hopkins, 2006). The global GW Eradication Program (GWEP), which was initiated in 1980 and led by the Carter Center, has reduced annual cases from an estimated 3.5 million human cases in Africa and Asia in 1986 (Watts, 1987) to 14 cases (Hopkins et al., 2024) in six sub-Saharan African countries—Angola, Chad, Ethiopia, Mali, Cameroon, and South Sudan—in 2023. GW is the first human parasitic disease targeted for eradication. The eradication effort is based on case containment and prevention of infection through health education and the promotion of water filters to prevent ingestion of copepods (Cairncross et al., 2002; Ruiz-Tiben and Hopkins, 2006). The disease primarily affects rural communities lacking access to clean water. Infected individuals remain asymptomatic throughout the 10–14-month prepatent period, after which a gravid adult female worm migrates to the subcutaneous tissues and produces a painful blister through which first-stage larvae (L1) are released upon contact with water (Ruiz-Tiben and Hopkins, 2006). Historically, treatment has been performed by gradual extraction and winding of the worm around a small stick—an extremely painful and debilitating process that can be complicated by secondary bacterial infection (Ruiz-Tiben and Hopkins, 2006; Cairncross et al., 2002).

Despite the success in reducing human cases, the emergence of GW in animals, especially domestic dogs, poses an additional challenge to the GWEP and may delay eradication. Although animal infections by GW were recorded previously, both naturally and experimentally, following the end of human transmission in endemic countries such as Uzbekistan, no animal infections were reported from those regions (Eberhard et al., 2016b; Molyneux and Sankara, 2017; Cairncross et al., 2002). At the beginning of 2011, rumors of emerging GW in dogs in Chad prompted the GWEP to include dogs in active village-based surveillance starting in April 2012. Several cases of GW in dogs were reported along the Chari River in Chad in 2012 (Eberhard et al., 2014; Guagliardo et al., 2020). The number of reported infections in dogs increased significantly due to active surveillance and the provision of cash rewards for reporting suspected cases (Hopkins et al., 2015). Furthermore, population genetic studies confirmed that the emerging worms from dogs were *D. medinensis* and that the same population of worms infects both humans and dogs (Thiele et al., 2018; Durrant et al., 2020). A total of 886 animal infections were reported in 2023, nearly 90% of them from dogs in Chad and Cameroon, followed by cat cases in Chad (Hopkins et al., 2024). Dogs in high-transmission areas can also become infected through ingestion of frogs as paratenic hosts or fish as transport hosts carrying infected copepods, in addition to ingesting infected copepods in drinking water (Cleveland et al., 2019, 2017; Eberhard et al., 2016a).

In the absence of an effective therapy or vaccine, GW eradication relies on containment or tethering of infected dogs and treatment of water sources with the organophosphate larvicide temephos (Abate^®^) to reduce copepod populations (Eberhard et al., 2014; Wang et al., 2023). However, given the logistical challenges of applying these methods and their variable effectiveness, new tools are needed for early detection of animal reservoirs to help the GWEP implement interventions before worm emergence and to increase the likelihood of interrupting transmission. These needs were reflected in the updated GWEP research agenda (Delea et al., 2024). Given the long prepatent period of 10–14 months for GW (Muller, 1971; Cairncross et al., 2002), refined diagnostic methods are needed to detect infected animals for containment or treatment if therapeutics become available in the future. Diagnostic tools will be essential to confirm the absence of GW transmission in animal reservoirs before endemic countries can be certified as GW-free. To identify immunoreactive antigens, GW crude proteins were screened using plasma samples from infected humans, and two proteins—thioredoxin-like protein 1 (TRX) and a domain of unknown function protein 148 (DUF148)—were identified (Priest et al., 2020). These antigens were further validated using a limited number of dog serum samples with recent GW infections from endemic areas in a multiplex bead assay (Priest et al., 2021). While these two antigens showed promising results in detecting antibodies against GW after worm emergence, they have not been assessed for detecting prepatent infections or evaluated for potential cross-reactivity with other parasitic nematodes.

In this study, we further evaluated these two GW antigens for accuracy in detecting GW infection using sera from experimentally infected dogs and a ferret, along with sera from Chadian dogs with a history of GW emergence. To determine possible cross-reactivity with other canine parasitic nematodes, we assessed these antigens using sera from shelter dogs in Texas, United States, a non-endemic area for GW, via indirect enzyme-linked immunosorbent assay (iELISA). We found that DUF148 was more reactive and capable of detecting prepatent infections but cross-reacted with several sera from US dogs. To mitigate DUF148 cross-reactivity, we produced shorter peptides and found that peptide 3 from the C-terminal region of the protein was more immunogenic and showed the best sensitivity for detecting GW infection. However, it did not improve the specificity of the iELISA compared with the whole antigen. Altogether, these results highlight the applicability and limitations of DUF148 for detecting prepatent GW infections in the field.

## 2 Materials and methods

### 2.1 Dog sera

#### 2.1.1 GW endemic area

From September 2021 to May 2023, serum samples were collected as part of a GW therapeutic trial in 56 villages along the Chari River from three regions (Moyen-Chari, Chari Baguirmi, and Mayo-Kebbi Est) in Chad. All procedures were conducted in accordance with the National Bioethics Committee of Chad (Protocol #005/PR/MESRI/SE/DGM/CNBT/SG/2022) and the University of Georgia Institutional Animal Care and Use Committee (A2019-04-005-Y4 A2). Initially, 1,210 dogs were enrolled in 2021, and more dogs were recruited in 2022, bringing the total to 2,495. Owner consent was obtained for all animals before enrollment and sample collection. Blood was collected from dogs via venipuncture by trained personnel and centrifuged to separate serum. Serum samples were frozen, shipped to Texas A&M University, and stored at −80 °C until testing. During this period, any GW emergence from enrolled dogs was recorded, and a worm sample was sent to the Centers for Disease Control and Prevention (CDC) for confirmation of GW per program guidelines.

#### 2.1.2 Non-endemic GW area

Matching blood and serum samples were collected from 492 shelter dogs in Brazos and Harris Counties, Texas, United States (IACUC 2022-0261). These samples were originally collected for the development of a probe-based quantitative PCR (qPCR) assay for heartworm (*Dirofilaria immitis*) diagnosis (Negron et al., 2022). Serum samples were screened for heartworm antigen using DiroCHEK^©^ (Zoetis Inc., Kalamazoo, MI, USA), a commercially available ELISA-based detection test (Sobotyk et al., 2021). Sera from 49 dogs with suspected or confirmed *Onchocerca lupi* infection were collected between January and September 2023 from New Mexico, United States, in another study developing diagnostics for this parasite (IACUC 2022-0261) (de Oliveira et al., 2020).

### 2.2 Experimental animal infections

Two dogs and one ferret were experimentally infected with GW. All animal work and experiments were conducted in accordance with the Animal Welfare Act, and the protocol was approved by the Texas A&M University Institutional Animal Care and Use Committee (IACUC AUP 2023-0273).

For dog infections, laboratory-reared copepods—descended from populations originally collected from Chad and identified as *Mesocyclops*, *Thermocyclops*, and *Eucyclops* based on partial *cytochrome oxidase subunit 1* gene sequences (Powers et al., 2020; Folmer et al., 1994), were exposed to L1 GW larvae recovered from *D. medinensis* females removed from an experimentally infected ferret at the University of Georgia (Eberhard et al., 1988, 2016). The exposed copepods were maintained for 2 weeks to allow GW larvae to molt to the infective L3 stage. Two laboratory-raised, spayed female beagles (Ridglan Farms, Inc., Mount Horeb, WI, USA) were each exposed *per os* to 40 copepods infected with GW L3 larvae. Dogs were group-housed and provided food and water ad libitum. Blood samples were collected from the saphenous vein before inoculation and then weekly after GW exposure. Whole-blood and serum samples were frozen at −80 °C until testing.

For ferret infection, copepods were exposed to L1 larvae from a gravid GW recovered from a naturally infected dog in Chad, and infected copepods were transferred to Texas A&M University. The copepods were monitored for 18 days until they were confirmed to contain L3 GW larvae. Copepods were dissected under a microscope to release L3 larvae. A single female ferret was exposed intraperitoneally to nine L3 larvae. Blood samples were collected before inoculation and then biweekly thereafter.

Serum samples from dogs experimentally infected with *Brugia malayi* (n = 6) and *B. pahangi* (n = 5) were obtained from the Filariasis Research Reagent Resource Center (FR3) (IACUC A2022-04-009). Additionally, 15 serum samples from dogs experimentally infected with gastrointestinal nematodes (GIN)—*Ancylostoma caninum* (n = 10), *Uncinaria stenocephala* (n = 2), *Toxascaris leonina* (n = 1), and *Toxocara canis* (n = 2)—were obtained from TRS Labs, Inc. (IACUC 24-01).

### 2.3 GW recombinant proteins production and peptides synthesis

Recombinant proteins were produced in *E. coli* by Biomatik (Canada). TRX and DUF148 are signal-peptide-containing proteins, and the recombinant proteins were expressed as mature forms without signal peptides. Recombinant proteins were expressed as tag-free and purified using size-exclusion chromatography. The lyophilized proteins were reconstituted in molecular-grade water at 0.5 mg/mL and stored at −80 °C until use.

Three peptides were synthesized by Biomatik, covering the full length of DUF148 with partial overlaps (indicated in bold): QFDDDIPPFLKGAPQSTIKEFETILQNGQSQTDQQLDA**NINAWIAKQTSA;** Peptide 2: **NINAWIAKQTSA**IQNAYRTFMAQIRTAQQQAEQARRTML**AKFSADARAAD;** Peptide 3: **AKFSADARAAD**AQLTKIAEDPRLTGEQKQAKIEATFKGSKLSNYNSRKCL. The peptides were reconstituted in DMSO at 5 mg/mL and stored at −80 °C until use.

### 2.4 ELISA optimization

Microplates (Nunc Maxisorp, Thermo Fisher Scientific, Rockford, USA) were coated with 0.1–1,000 ng of each GW recombinant protein (TRX and DUF148) or DUF148 peptides to determine the optimal concentration. Following initial optimization, plates were coated overnight at 4 °C with 500 ng of GW proteins or 200 ng of DUF148 peptides in 100 µL of coating buffer (50 mM carbonate/bicarbonate buffer, pH 9.6). The wells were blocked with 300 µL of 5% skimmed milk in phosphate-buffered saline with 0.05% Tween 20 (PBS-T). Antigen-coated wells were then filled with diluted serum and incubated overnight at 4 °C.

Test sera (100 µL) were diluted 1:1,000 in PBS-T containing 5% skimmed milk, and the secondary antibody (100 µL) was diluted 1:10,000 and incubated at 37 °C for 1 h. Horseradish peroxidase (HRP)-conjugated rabbit anti-dog IgG (Sigma, A6792) or goat anti-ferret IgG (Novus Biologicals, NB7224) was used as the secondary antibody. Optical density (OD) was measured at 450 nm using a Synergy H1 microplate reader (Biotek, Winooski, VT, USA). All specimens were tested in duplicate.

### 2.5 Double centrifugal fecal floatation

Fecal samples were collected from shelter dogs at the time of blood sampling. Double-centrifugal fecal flotation was performed using Sheather’s sugar solution as described by (Zajac et al., 2021) with minor modifications, to detect gastrointestinal parasites. Briefly, 2 g of feces was measured into a clean disposable cup, mixed with water, strained through cheesecloth, and transferred to a 15 mL tube. After centrifugation at 400 × g for 10 min, the supernatant was removed, and sugar solution was added to a final volume of 10 mL. The fecal pellet was thoroughly mixed, and the tubes were centrifuged again at 400 × g for 10 min. Sugar solution was then added to the tubes to form a positive meniscus. A coverslip was placed on top and the tube was incubated for an additional 10 min. The coverslip was carefully lifted, placed on a glass slide, and observed under a microscope to identify parasite eggs.

### 2.6 Statistical analysis

The iELISA sensitivity, specificity, and optimal density cut-off were calculated by plotting receiver operating characteristic (ROC) curves using Prism. To test for statistical significance between the mean OD values of GIN-positive and GIN-negative sera, a Mann–Whitney *U* test was performed. Differences were considered statistically significant at *P* < 0.05.

## 3 Results

### 3.1 Optimization of GW antigen concentration for indirect ELISA (iELISA)

To determine the optimal antigen concentration for iELISA, we used serum samples from Chadian dogs with a history of GW emergence, shelter dogs from a non-endemic GW area (Texas, USA), and naïve, purpose-bred, laboratory-reared beagle dogs. Plates were coated with 0.1–1,000 ng of each GW recombinant protein (TRX and DUF148) or a cocktail of both proteins (1:1). We found that 500 ng of each antigen provided the best optical density (OD) separation between positive and negative sera (**Supplementary Figure S1**), and this concentration was used for coating in subsequent experiments.

### 3.2 iELISA of experimentally infected dogs and ferret with GW

**Figure 1:**
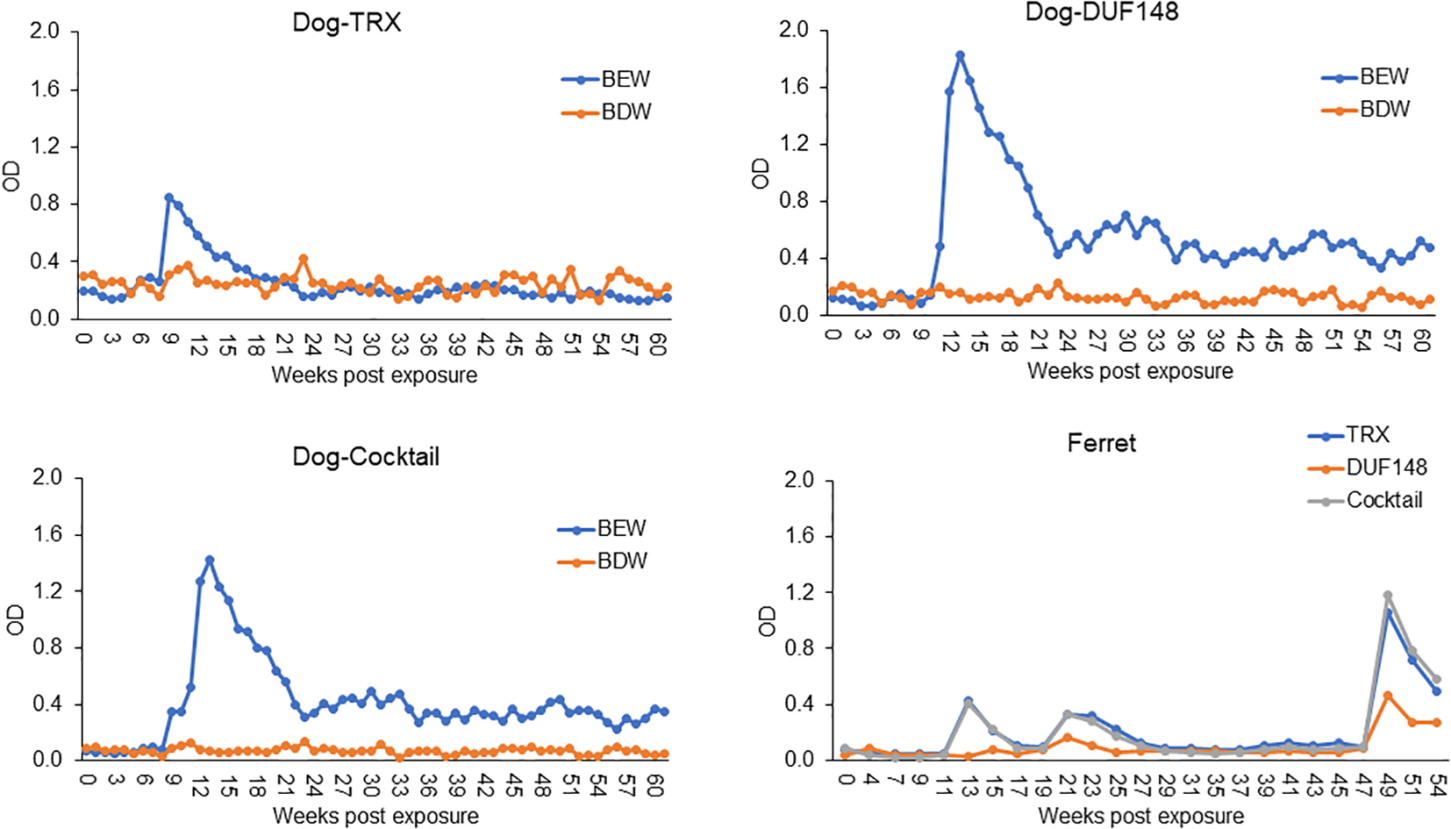
Time-course reactivity of weekly (dogs) or biweekly (ferret) collected sera in TRX, DUF148, or cocktail iELISA. Dogs (BEW and BDW) were exposed to copepods infected with third-stage larvae (L3), and the ferret was exposed to L3 larvae at time point 0.

We used the iELISA with each GW antigen or a cocktail of both antigens to screen sera from two dogs (BEW and BDW) and a ferret experimentally infected with GW (**Figure 1**). The dogs were exposed by oral gavage with copepods infected with L3 larvae, and the ferret was exposed intraperitoneally to L3 larvae isolated from infected copepods. Sera from BEW showed increased reactivity at 9 and 11 weeks post-exposure to TRX and DUF148, respectively. However, titers to both antigens gradually declined over 14 months post-exposure, when necropsy was performed. No GW was retrieved from these dogs, indicating that infection did not establish. Sera from BDW showed no reactivity to GW antigens, and no GW was retrieved during necropsy. Ferret sera showed increased reactivity to TRX and DUF148 at 13 and 15 weeks post-exposure, respectively (**Figure 1**). Titers decreased gradually but showed two additional peaks at 21 and 49 weeks post-exposure. Prepatent infection was confirmed by the detection of a subcutaneous worm via ultrasound at 9 months post-exposure (**Supplementary Figure S2**). Seven gravid female nematodes containing L1 larvae were retrieved from the ferret during necropsy at 12 months post-exposure (**Supplementary Table S1**).

### 3.3 Evaluation of iELISA for detection of prepatent GW infection in Chad dogs

To assess the performance of DUF148 and TRX iELISA for detecting GW infection, archived sera from Chadian dogs with a history of GW emergence were selected. Considering the 10–14-month prepatent period (Cairncross et al., 2002) and an estimated 4-month interval between ingestion of infected copepods and the appearance of antibodies in dogs based on our experimental infections, we selected 47 serum samples collected within 6 months prior to reported GW emergence. Sera from 492 US shelter dogs served as negative controls from a non-endemic area.

**Figure 2:**
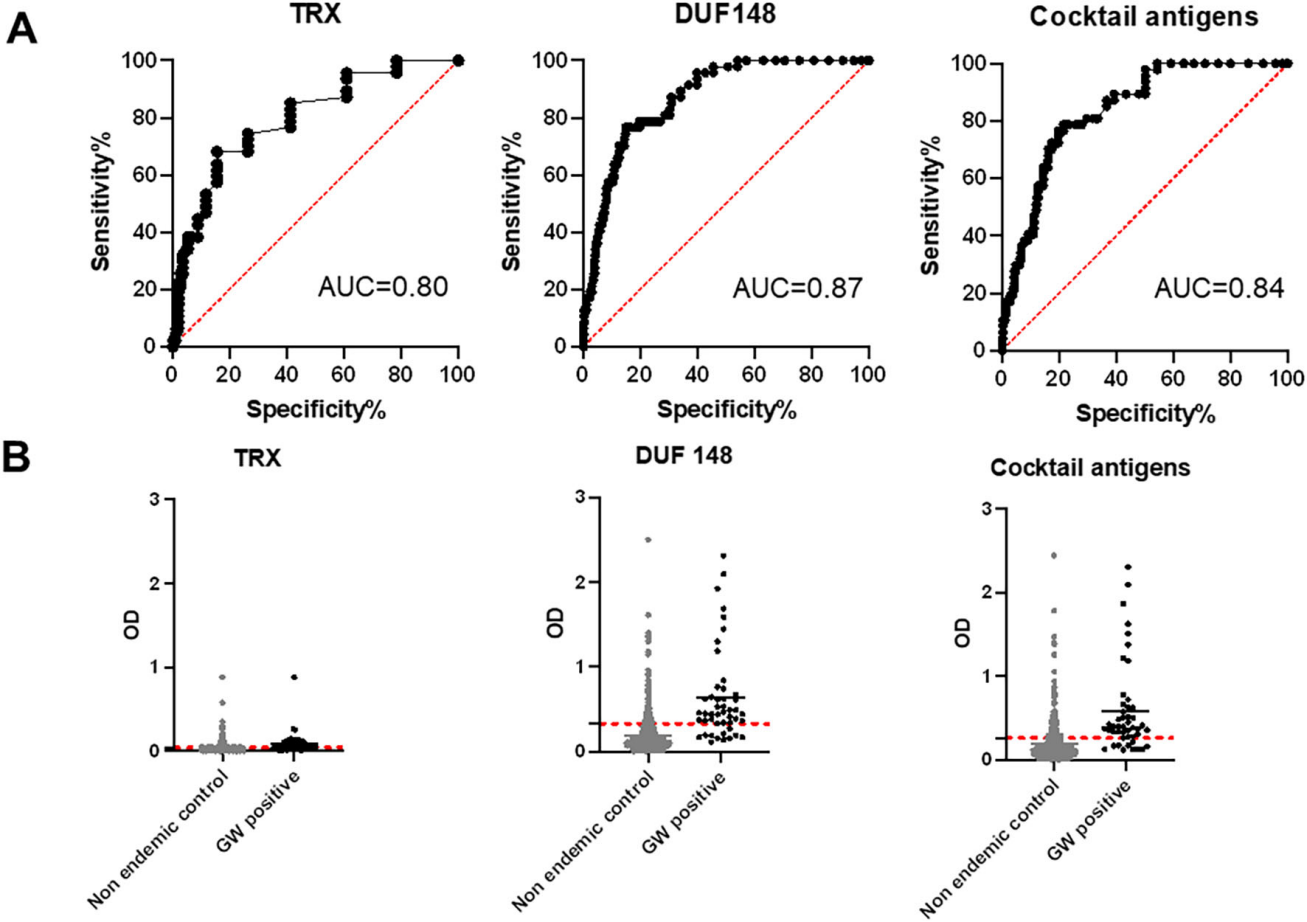
ROC analysis and reactivity of dog sera to GW antigens. **(A)** ROC analysis was performed to define the cut-off value, area under the curve (AUC), sensitivity, and specificity using 47 positive (Chad) and 492 negative (United States) dog sera. **(B)** Dashed lines represent the cut-off (determined by ROC curves) in absorbance level.

Receiver operating characteristic (ROC) analyses were performed to estimate the cut-off and performance indices, namely the area under the curve (AUC), sensitivity, specificity, positive predictive value (PPV), and negative predictive value (NPV) for DUF148, TRX, and the antigen cocktail (**Figure 2**; **Table 1**). DUF148 was more antigenic and showed better performance than TRX, with 76.6% sensitivity (39/47), 85.2% specificity, and an AUC of 0.87. The antigen cocktail showed higher sensitivity (78.7%) but lower specificity (78.5%).

**Table 1:** Diagnostic performance of GW antigens and DUF148 peptides.

Target Antigen	AUC	CI (95%)	Cutoff OD	Sensitivity (%)	Specificity (%)	PPV (%)	NPV (%)
TRX	0.80	0.73 - 0.86	> 0.04025	68.1	84.4	68.1	80.6
DUF148	0.87	0.83 – 0.91	> 0.3313	76.6	85.2	76.6	86.5
Cocktail antigen	0.84	0.79 – 0.89	> 0.2608	78.7	78.5	78.7	80.1
Peptide 1	0.60	0.51 – 0.69	< 0.09775	59.6	63.2	ND	ND
Peptide 2	0.81	0.75 – 0.87	< 0.06825	78.7	74.2	ND	ND
Peptide 3	0.84	0.79 – 0.89	> 0.1523	83	71.5	83	74.4
Cocktail of Peptides 1&3	0.84	0.80 – 0.89	> 0.1003	85.1	69.3	85.1	69.8

AUC, area under the curve; CI, confidence interval; PPV, positive predictive value; NPV, negative predictive value; ND, not determined.

**Figure 3:**
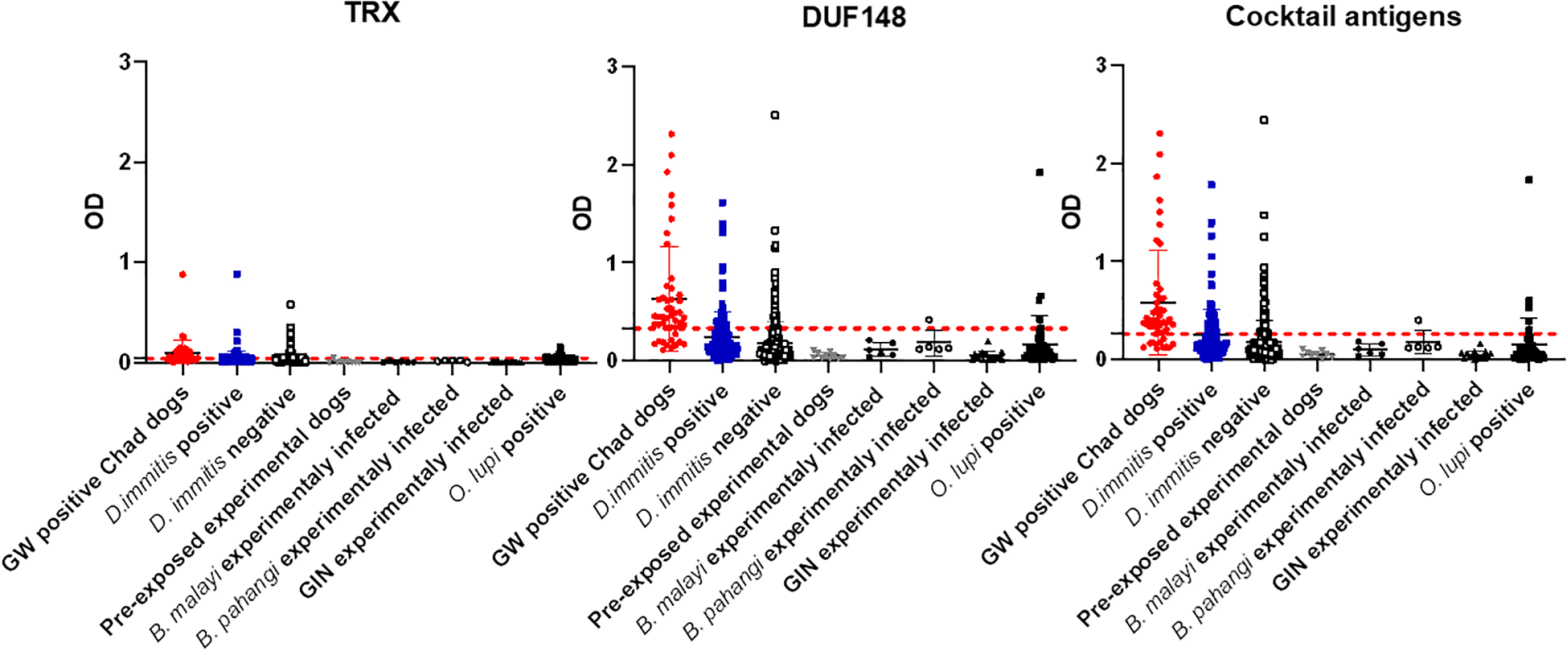
Optical density (OD) values for GW antigens among different groups of dogs. Mean OD values for each group are shown. Dashed lines represent the cut-off in absorbance level determined by ROC curves. US shelter dogs are divided into *Dirofilaria immitis*-positive and -negative groups. *D. immitis* and *Onchocerca lupi* represent natural infections.

However, 73 (14.8%) and 97 (19.7%) US dog sera cross-reacted with DUF148 and TRX, respectively. These dogs were screened for heartworm (*Dirofilaria immitis*) using various tests (de Oliveira et al., 2020), and both TRX and DUF148 cross-reacted regardless of heartworm status (**Figure 3**). TRX and DUF148 are signal-peptide-containing proteins predicted to be secreted by GW and are conserved across the phylum Nematoda with considerable sequence similarity (**Supplementary Figure S3**).

**Figure 4:**
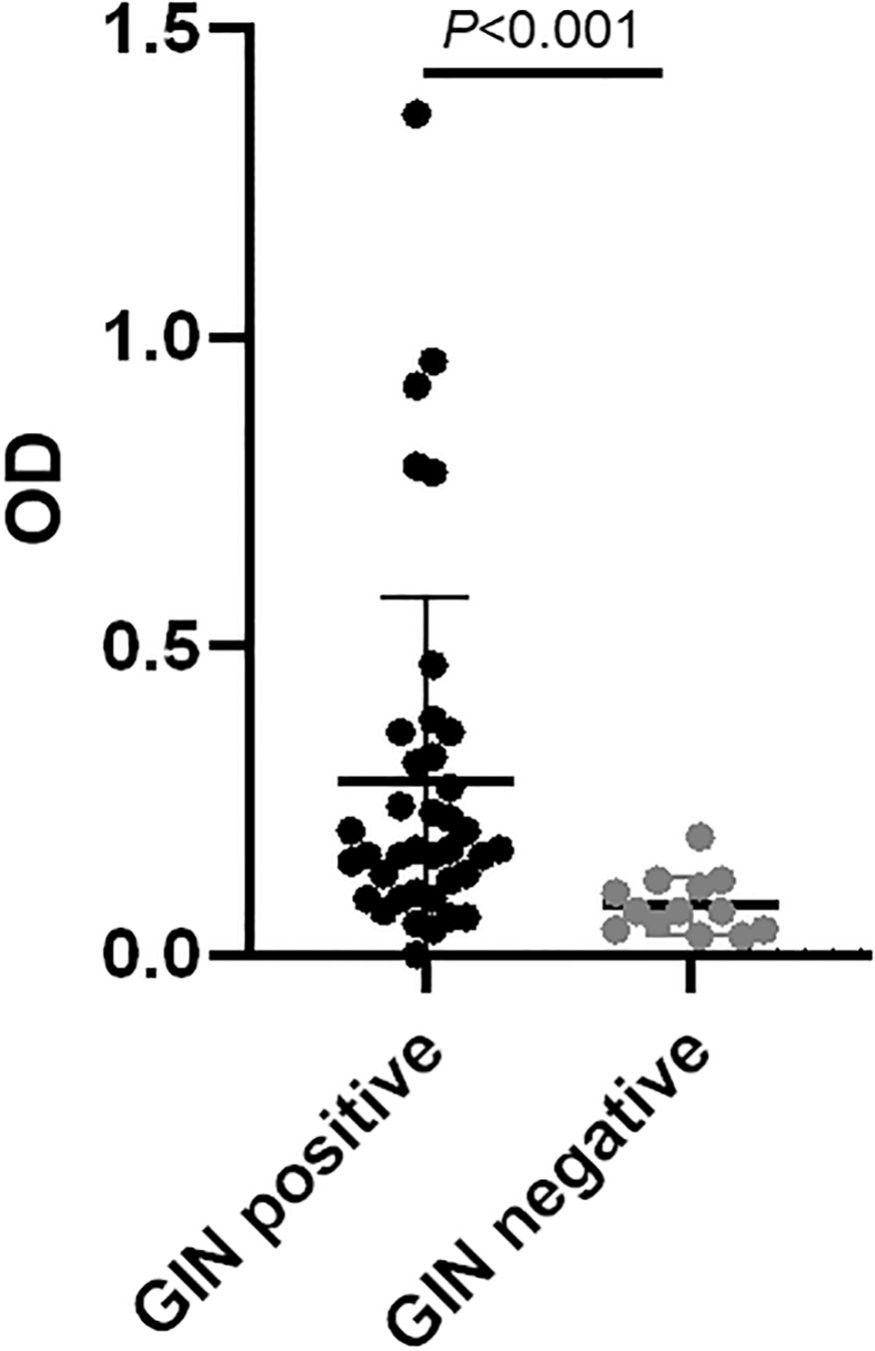
Reactivity of US shelter dog sera positive or negative for gastrointestinal nematodes (GIN) with DUF148. Thirty-eight fecal samples from shelter dogs were positive and 13 were negative for GIN eggs using the double-centrifugal sugar flotation test. The *P* value was determined by the Mann–Whitney *U* test.

We further screened DUF148 reactivity with sera from experimentally infected dogs with *Brugia pahangi*, *B. malayi*, and gastrointestinal nematodes (GIN; *Ancylostoma caninum*, *Toxocara canis*, *Toxascaris leonina*, and *Uncinaria stenocephala*). Cross-reactivity was observed in one *B. pahangi*-infected dog. We also assessed cross-reactivity using shelter dog sera with paired fecal test results (double-centrifugal sugar flotation) confirming GIN infection (**Supplementary Table S2**). DUF148 reacted significantly more with sera from GIN-positive dogs than with GIN-negative dogs (**Figure 4**). Additionally, we screened archived sera from US dogs naturally infected with the filarial nematode *Onchocerca lupi* for cross-reactivity with GW antigens. DUF148 cross-reacted with 4 samples, whereas TRX cross-reacted with 14 out of 49 samples (**Figure 3**). Overall, these results indicate that GW antigens cross-reacted with sera from *B. pahangi*-positive, GIN-positive, and *O. lupi-*positive dogs.

### 3.4 DUF148 peptide iELISA

To mitigate cross-reactivity, we synthesized three short peptides from DUF148 that covered the entire mature DUF148 antigen, with each peptide partially overlapping the others. The optimal concentration of each peptide for iELISA was determined (**Supplementary Figure S4**).

**Figure 5:**
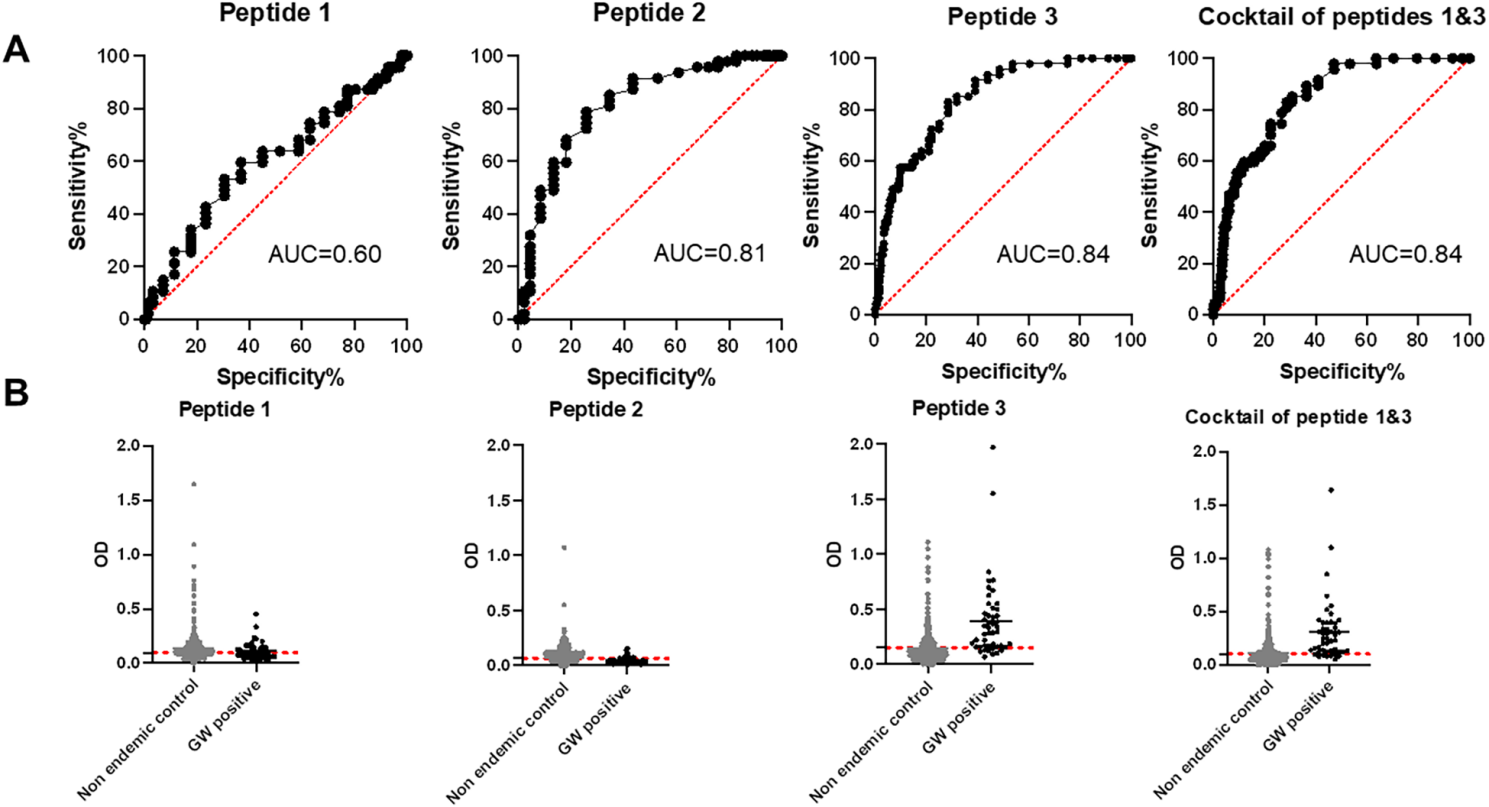
ROC analysis and reactivity of dog sera to DUF148 peptides. **(A)** ROC analysis was performed using 47 positive (Chad) and 492 negative (United States) dog sera to define the cut-off value, area under the curve (AUC), sensitivity, and specificity. **(B)** Dashed lines represent the cut-off in absorbance level.

**Figure 6:**
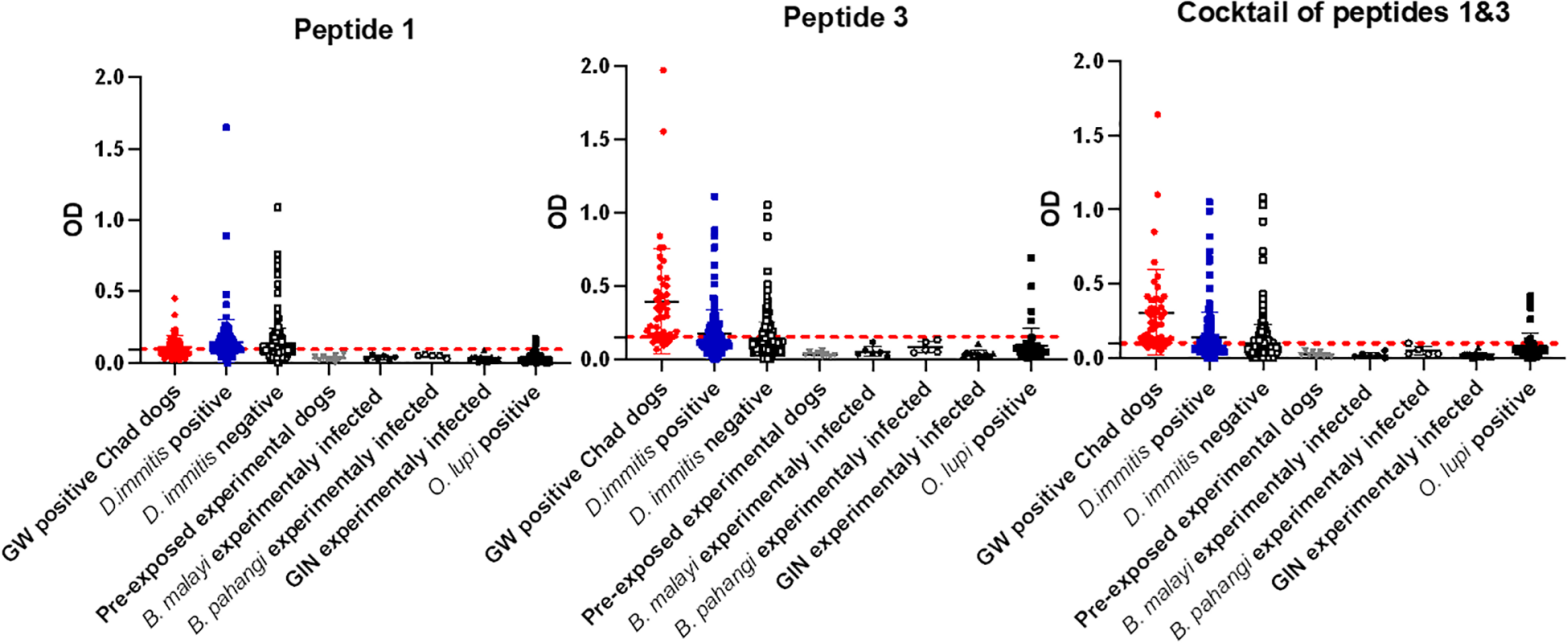
Optical density (OD) values for DUF148 peptides among different groups of dogs. Mean OD values for each group are shown. Dashed lines represent the cut-off in absorbance level. US shelter dogs are divided into *D. immitis* antigen-positive and -negative groups. *D. immitis* and *O. lupi* represent natural infections.

Initial screening showed that peptide 2, corresponding to the middle portion of the protein, was not immunoreactive to GW-positive sera. Therefore, we proceeded with peptide 1, peptide 3, and a cocktail of peptides 1 and 3. Peptide 1 (the N-terminal region of DUF148) was less reactive to GW-positive sera than to dog sera from the non-endemic United States (**Figure 5**; **Table 1**). Peptide 3, which encompasses the C-terminal region of DUF148, showed the best performance (**Figure 5**; **Table 1**), with a sensitivity of 83%, specificity of 71.5%, and an AUC of 0.84 compared with peptide 1. It did not cross-react with sera from experimentally infected dogs with *Brugia* spp. or gastrointestinal nematodes (GIN) (**Figure 6**). However, cross-reactivity was observed with sera from US shelter dogs and *Onchocerca lupi*-positive dogs.

The cocktail of peptides 1 and 3 showed higher sensitivity (85.1%) but lower specificity (69.3%) compared with peptide 3 alone (**Table 1**). Overall, peptide 3 demonstrated greater sensitivity than the whole DUF148 antigen, although specificity and AUC did not improve.

### 3.5 iELISA of Chad dog sera pre- and post GW emergence

**Figure 7:**
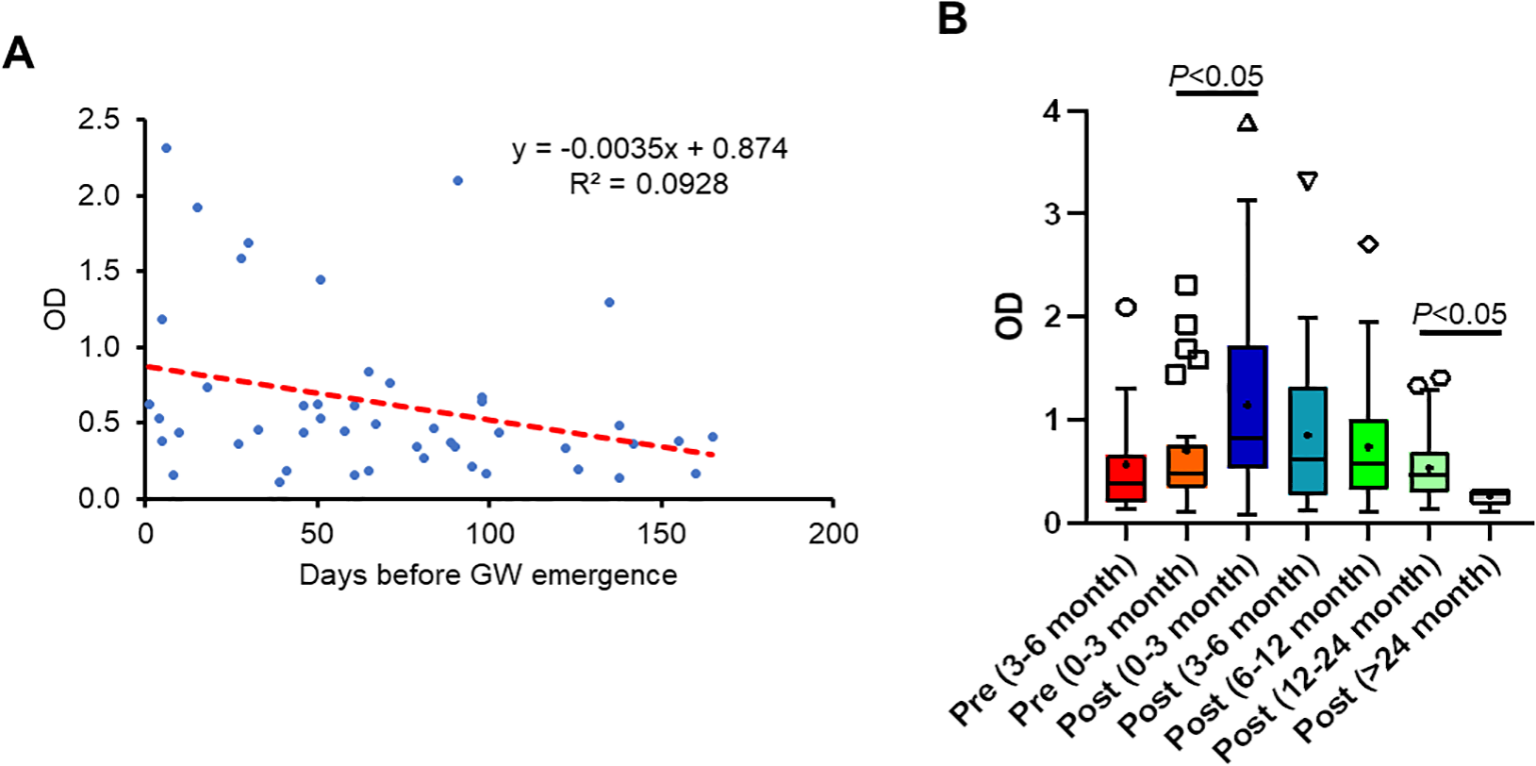
Correlation between antibody response to DUF148 and the time of GW emergence. **(A)** DUF148 titers of dog sera (n = 47) plotted against days prior to GW emergence. The dashed line shows the linear correlation. GW emergence time is shown as 0 on the x-axis. **(B)** Box plots denote medians and interquartile ranges (whiskers denote the median ± 1.5 × interquartile range) of different groups of dog sera (n = 212) with GW emergence. *P* values were determined by the Mann–Whitney *U* test.

To determine whether there was a correlation between antibody response to GW antigens and the time before GW emergence, we examined DUF148 reactivity relative to the number of days before worm emergence. Although an increasing trend in DUF148 titer was observed as the day of worm emergence approached, there was no significant correlation between DUF148 titer and days pre-GW emergence (**Figure 7A**).

Because antibody titers wane over time, we assessed the duration of anti-DUF148 antibody levels in dog sera with a single infection collected during the 6 months before GW emergence up to 2 years after emergence. Dogs with multiple GW emergence events during the sampling period were defined as having a single infection if the worms emerged within 1 month. The highest titers were observed during the first 3 months post-GW emergence, gradually decreasing thereafter but remaining detectable up to 2 years post-emergence (**Figure 7B**).

## 4 Discussion

Development of reliable diagnostic methods for detecting GW infection in reservoir animals is essential to support the global GWEP, enabling targeted containment and treatment of exposed animals as therapeutic candidates become available. In this study, we assessed two GW antigens, TRX and DUF148, in an iELISA format that could be used for diagnosing prepatent GW infection in dogs. Previous work with these GW antigens was performed in a multiplex bead assay format that relied on GST-tagged antigens and monoclonal anti-canine IgG4 antibodies (Priest et al., 2020, 2021). GST (glutathione S-transferase) is derived from *Schistosoma* and used for purification of recombinant proteins (Smith and Johnson, 1988). TRX and DUF148 contain signal peptides, indicating that they are likely secreted by GW. In our iELISA, we used mature, tag-free proteins to reduce the likelihood of cross-reactivity with other helminths, which could generate false-positive results and compromise assay specificity. We also used a commercial polyclonal anti-dog IgG secondary antibody instead of targeting canine IgG4, as IgG4 in humans comprises about 4% of bulk IgG and tends to appear later in chronic infections (Rispens and Huijbers, 2023) potentially affecting assay sensitivity.

In experimental animals, specific antibodies against TRX were detected as early as 9 weeks post-exposure in dogs and 13 weeks in the ferret. Antibody titers against DUF148 appeared approximately 2 weeks later in both animals, indicating different timing in antigen release by the parasite. In this study, neither experimentally exposed dog developed a patent infection—that is, no subcutaneous gravid female worms emerged. We believe these immunocompetent dogs controlled GW development at an early stage. Therefore, implementing immunosuppression during exposure and early parasite development may be necessary to achieve patent infections in future experimental trials. The WHO developed a target product profile for a serological test to detect prepatent GW infections in animals, specifying a minimum sensitivity of 80% and specificity of 90% (World Health Organization, 2024). The current sensitivity and specificity of our DUF148 iELISA—76.6% and 85.2%, respectively—are promising but still below the target. Our effort to use shorter peptides to mitigate cross-reactivity improved sensitivity to 83% but did not improve specificity.

We believe the cross-reactivity observed in non-GW-endemic samples results from DUF148 being a conserved antigen across Nematoda. We attempted to attribute this cross-reactivity to specific parasites and found that exposure to *Brugia* spp., *O. lupi*, and GIN was significantly associated with false-positive results on the DUF148 iELISA, whereas heartworm infection was not. *Dracunculus insignis*, a common parasite of raccoons, has been reported in dogs in Texas (Williams et al., 2018) and could be another contributing factor to the cross-reactivity observed in US dog sera with DUF148. Our findings align with published reports showing that cross-reactivity of human sera to DUF148 has been attributed to infections with *Onchocerca volvulus*, *Wuchereria bancrofti*, and *Loa loa* (Priest et al., 2020). Given these observations, the recent detection of *Brugia* sp. in Chadian dogs (Haynes et al., 2022), and the global distribution of GIN, DUF148 may not be the most specific antigenic candidate for a GW serological diagnostic method, particularly for screening naturally infected dogs in GW-endemic areas.

When evaluating the assay’s potential as a prepatent diagnostic, we found no correlation between antibody titers against DUF148 and days before GW emergence. Based on the antibody response to GW in the experimentally infected ferret, it appears that antibody titers fluctuate during the prepatent period, likely reflecting GW migration through subcutaneous tissues. The highest antibody titers in the infected ferret were observed when subcutaneous worms became visible and approached emergence, suggesting that the immune response was associated with local tissue damage caused by GW migration. In clinical specimens, titers significantly increased following GW emergence in Chadian dogs, likely due to local inflammation and blister formation at the emergence site. Although antibody responses waned gradually over time, anti-DUF148 antibodies were still detectable up to 2 years post-emergence. Several factors may contribute to this prolonged antibody persistence, including infection intensity, host immune status, and possible secondary exposure during follow-up.

In the absence of diagnostic methods for GW infection, the DUF148 iELISA could serve as a starting point for detecting and managing infections in dogs. Because antibody-detection assays such as iELISA identify host immune responses, they may not differentiate between active and past infections. However, the intended use of the assay is as a screening tool within an integrated surveillance strategy, not as a sole diagnostic for eradication certification or treatment decisions. TRX and DUF148 are valuable targets for monitoring experimentally infected animals under laboratory conditions. However, more specific serological markers are needed for field application in GW-endemic regions. This may be achieved using comprehensive methods to identify serological markers based on the GW proteome. PepSeq technology, which builds peptide libraries from the organism’s proteome (Henson et al., 2023), would allow comprehensive screening of immunogenic linear peptides of GW. Following the discovery of novel immunogenic targets, unique antigens with improved performance in detecting prepatent GW infection should be prioritized to meet WHO’s target product profile. Overall, our data support the value of GW serological tests for monitoring host antibody responses and surveillance of GW exposure in animals residing in endemic regions, thereby supporting continued optimization and validation of serological diagnostics for GW disease.

## Data Availability

The original contributions presented in the study are included in the article/**Supplementary Material**. Further inquiries can be directed to the corresponding authors.
