# Supplementary material for: Assessing the performance of TRX and DUF148 antigens for detection of prepatent Guinea worm (Dracunculus medinensis) infection in dogs
Source: Front Parasitol. 2025 Nov 21;4:1699367. doi: 10.3389/fpara.2025.1699367 (PMC12678139; doi:10.3389/fpara.2025.1699367)
Supplement: Supplementary file 1 [file DataSheet1.docx]

Supplementary Material


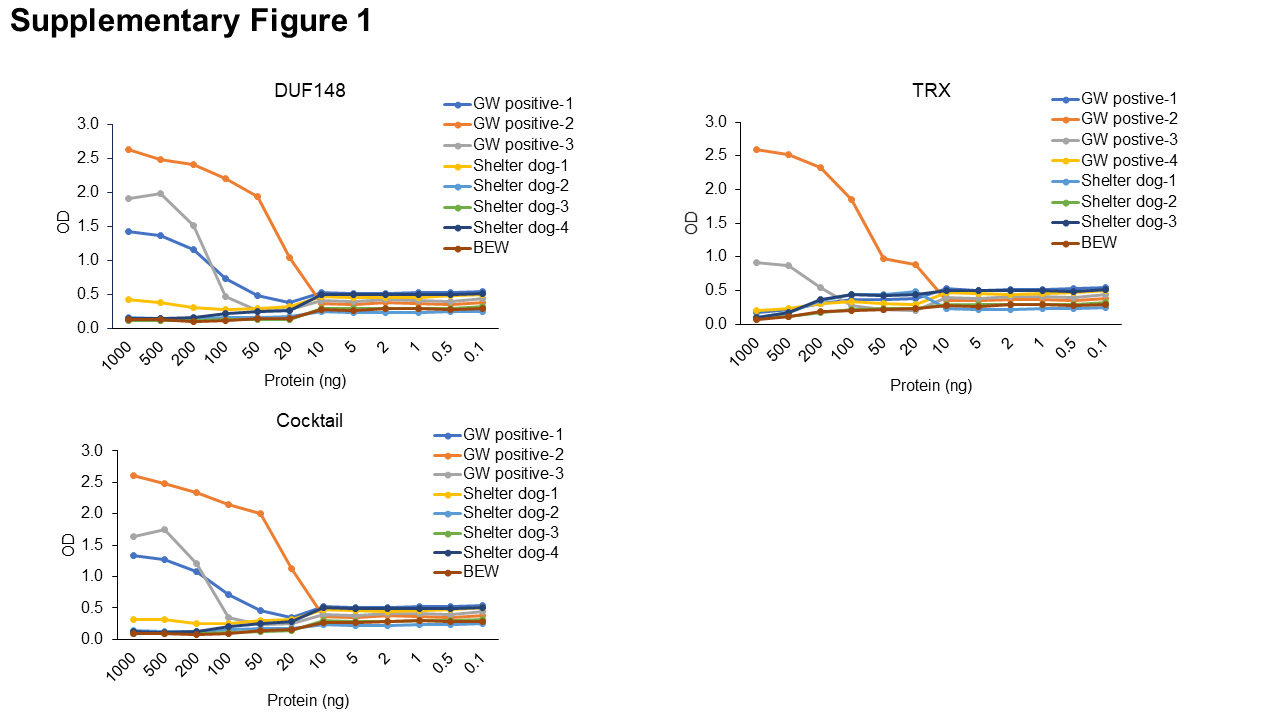


**Supplementary Figure 1. Optimization of GW antigen concentration for iELISA.** GW positive or negative (US shelter dogs and pre-immune sera from experimental dog, BEW) dog sera reactivity with different antigen concentrations (0.1-1000 ng) are plotted.


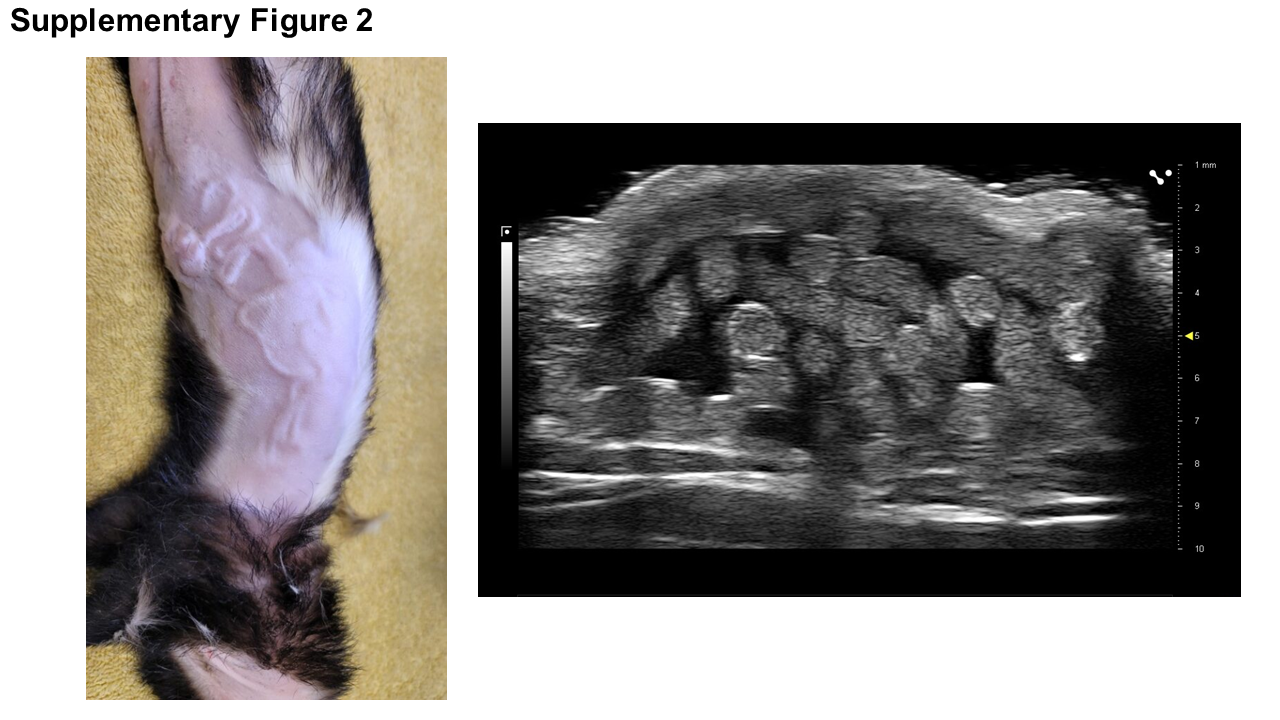


**Supplementary Figure 2. Experimental GW infection of ferret.** Subcutaneous worm was observed on the right side of ferret and GW was confirmed using ultrasound imaging at 9 months post exposure.


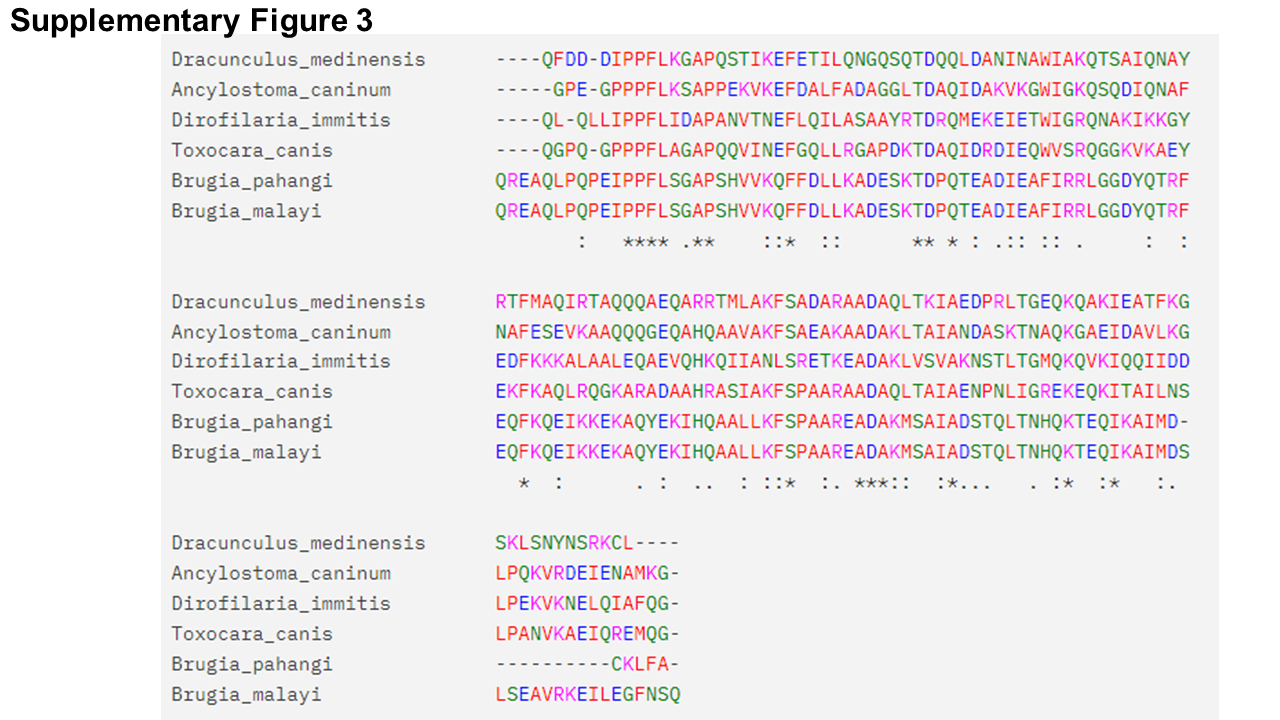


**Supplementary Figure 3. Multiple amino acid sequence alignment of GW DUF148 with its orthologs of other nematodes.** Asterisks show the conserved amino acids.


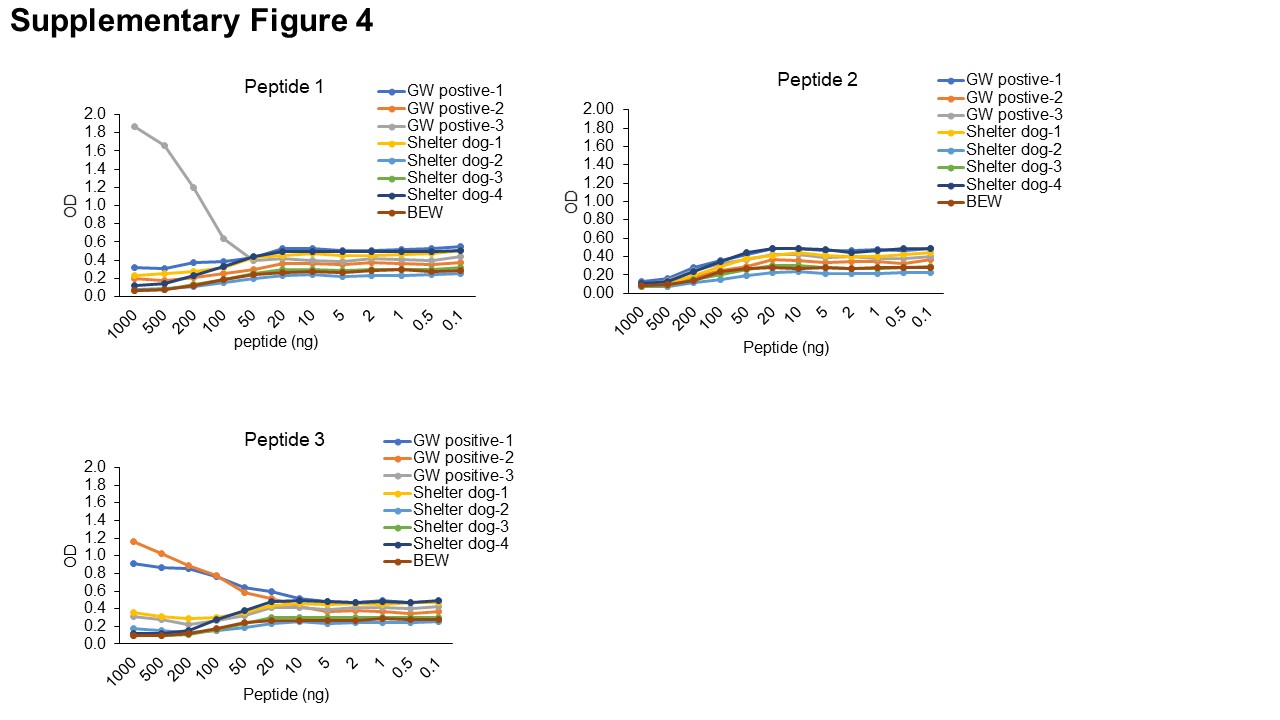


**Supplementary Figure 4. Optimization of DUF148 peptides concentration for iELISA.** GW positive or negative (US shelter dogs and pre-immune sera from experimental dog, BEW) dog sera reactivity with different antigen concentrations (0.1-1000 ng) are plotted.

**Supplementary Table 1. GW female specimens retrieved from ferret at necropsy**

|  | Length (cm) | Total L1* | Viability of L1 (%) | Comment |
| --- | --- | --- | --- | --- |
| GW1 | 69 | 384,000 | 0 | encapsulated |
| GW2 | 74 | 44,000 | 0 | encapsulated (umbilical) |
| GW3 | 112 | 192,000 | 39 | subcutaneous left rear leg |
| GW4 | 107 | 960,000 | 4.8 | encapsulated |
| GW5 | 94 | 0 | ND | encapsulated |
| GW6 | 84 | 0 | ND | encapsulated |
| GW7 | 91 | 800,000 | 100 | submandibular |

* Total L1 are the larvae that were released naturally from GW following transfer to water.

**Supplementary Table 2. Summary of double centrifugal fecal floatation**

| GIN positive | | | | | |
| --- | --- | --- | --- | --- | --- |
| Shelter dog | *Cystoisospora* (OPG) | *Ancylostoma* (EPG) | *Toxocara* (EPG) | *Trichuris* (EPG) | *Giardia* |
| 1 |  | >500 |  | 71.5 |  |
| 2 |  | >500 |  |  |  |
| 3 |  |  |  | 6.5 |  |
| 4 |  | 271 |  | 120 |  |
| 5 |  | >500 |  | >500 |  |
| 6 | 869 |  |  | 411 |  |
| 7 |  |  |  | 3 |  |
| 8 |  | 151 |  |  |  |
| 9 | 73 | 46 |  | 17 |  |
| 10 |  | 241 |  |  |  |
| 11 |  | >500 |  | 29 |  |
| 12 |  | >500 |  |  |  |
| 13 |  | >500 |  |  |  |
| 14 |  | 135.5 |  | 12 |  |
| 15 | *C. ohioensis* (11) | 118 |  |  |  |
| 16 |  | 122 |  |  |  |
| 17 | *C. ohioensis* (29.5) | 4 |  |  |  |
| 18 |  | 257.5 |  |  |  |
| 19 |  | >500 |  | 15.5 |  |
| 20 |  | 68 |  |  |  |
| 21 | *C. ohioensis* (221.5) | 125.5 |  |  |  |
| 22 |  | >500 |  |  |  |
| 23 |  | >500 | >500 |  |  |
| 24 | *C. canis* (8) | >500 |  |  |  |
| 25 | *C. ohioensis* (6) | 228.5 |  | 226.5 |  |
| 26 |  | >500 |  |  |  |
| 27 |  | 41 |  | 66.5 |  |
| 28 |  | 95.5 | >500 | >500 |  |
| 29 |  | 98.5 |  |  |  |
| 30 |  |  |  | 153 |  |
| 31 |  | 23.5 |  |  |  |
| 32 |  | 174 |  | 9.5 |  |
| 33 |  | >500 |  |  |  |
| 34 |  | >500 |  | 43 |  |
| 35 |  | >500 |  | >500 |  |
| 36 |  | >500 |  |  |  |
| 37 |  | 160.5 |  | 8 |  |
| 38 |  | >500 |  | 89 |  |
| GIN negative | | | | | |
| 1 | No Parasite Seen | | | | |
| 2 | No Parasite Seen | | | | |
| 3 | No Parasite Seen | | | | |
| 4 | No Parasite Seen | | | | |
| 5 | No Parasite Seen | | | | |
| 6 | No Parasite Seen | | | | |
| 7 | No Parasite Seen | | | | |
| 8 | No Parasite Seen | | | | |
| 9 | No Parasite Seen | | | | |
| 10 | No Parasite Seen | | | | |
| 11 | No Parasite Seen | | | | |
| 12 | No Parasite Seen | | | | |
| 13 | No Parasite Seen | | | | |

GIN, gastrointestinal nematode; OPG, oocyst per gram; EPG, egg per gram
